# Activation of FXR pathway does not alter glial cell function

**DOI:** 10.1186/s12974-017-0833-6

**Published:** 2017-03-28

**Authors:** Stefanie Albrecht, Ann-Katrin Fleck, Ina Kirchberg, Stephanie Hucke, Marie Liebmann, Luisa Klotz, Tanja Kuhlmann

**Affiliations:** 1 Institute of Neuropathology, University Hospital Münster, 48149 Münster, Germany; 2 Department of Neurology, University of Münster, 48149 Münster, Germany

**Keywords:** FXR, Nuclear receptors, Oligodendrocytes, Microglia, Astrocytes, GW4064

## Abstract

**Background:**

The nuclear receptor farnesoid-X-receptor (FXR; NR1H4) is expressed not only in the liver, gut, kidney and adipose tissue but also in the immune cells. FXR has been shown to confer protection in several animal models of inflammation, including experimental autoimmune encephalomyelitis (EAE), an animal model of multiple sclerosis (MS). FXR agonists are currently tested in clinical trials for treatment of human metabolic diseases. The beneficial effect of FXR agonists in EAE suggests that FXR might represent a potential target in inflammatory-demyelinating CNS diseases, such as MS. In MS, oligodendrocytes not only undergo cell death but also contribute to remyelination. This repair mechanism is impaired due to a differentiation block of oligodendroglial progenitor cells. Activation of other nuclear receptors that heterodimerize with FXR promote oligodendroglial differentiation. Therefore, we wanted to address the functional relevance of FXR for glial cells, especially for oligodendroglial differentiation.

**Methods:**

We isolated primary murine oligodendrocytes from FXR-deficient (FXR Ko) and wild-type (WT) mice and determined the effect of FXR deficiency and activation on oligodendroglial differentiation by analysing markers of oligodendroglial progenitor cells (OPCs) and mature oligodendrocytes (OLs) using qRT-PCR and immunocytochemistry. Additionally, we determined whether FXR activation modulates the pro-inflammatory profile of astrocytes or microglia and whether this may subsequently modulate oligodendroglial differentiation. These in vitro studies were complemented by histological analyses of oligodendrocytes in FXR Ko mice.

**Results:**

FXR is expressed by OPCs and mature oligodendrocytes. However, lack of FXR did not affect oligodendroglial differentiation in vitro or in vivo. Furthermore, activation of FXR using the synthetic agonist GW4064 did not affect oligodendroglial differentiation, remyelination in an ex vivo model or the expression of pro-inflammatory molecules in astrocytes or microglia. Concordantly, no effects of supernatants from macrophages cultured in the presence of GW4064 were observed regarding a possible indirect impact on oligodendroglial differentiation.

**Conclusions:**

Our data suggest that FXR is dispensable for oligodendroglial differentiation and that FXR agonists, such as GW4064, represent a potential therapeutic approach for MS which specifically targets peripheral immune cells including macrophages but not brain-resident cells, such as oligodendrocytes, astrocytes or microglia.

**Electronic supplementary material:**

The online version of this article (doi:10.1186/s12974-017-0833-6) contains supplementary material, which is available to authorized users.

## Background

Nuclear receptors are a large group of transcription factors that comprises 49 family members which enable the organism to quickly adapt to environmental changes by inducing the appropriate gene program [1]. Nuclear receptors are activated by lipophilic ligands including hormones, fatty acids, bile acids and oxysterols, homo- and heterodimerize upon activation and regulate the metabolism of glucose, fatty acids, triglycerides and lipoproteins [1]. Besides these metabolic effects, numerous immune-modulatory effects by nuclear receptors have been described, including modulation of central nervous system (CNS) autoimmunity [2–4].

Interestingly, several members of the nuclear receptor family are also involved in molecular mechanisms regulating oligodendroglial differentiation. Downregulation of retinoid X receptor gamma (RXRγ) expression or application of RXRγ antagonists significantly impaired oligodendroglial differentiation. Similarly, mice lacking RXRγ showed delayed remyelination after induction of demyelination in vivo [5]. In oligodendrocytes, RXRγ heterodimerizes with the vitamin D receptor (VDR) to induce oligodendroglial differentiation, and accordingly, vitamin D-induced activation accelerates oligodendroglial differentiation [6]. Other nuclear receptors, such as thyroid receptors (TR) and liver X receptors (LXR), are well expressed in oligodendroglial lineage cells and modulate oligodendroglial differentiation and myelination. Patients with congenital hypoparathyroidism typically develop a hypomyelinating phenotype, and triiodothyronine (T3) is required for the maturation of oligodendroglial progenitor cells into mature myelinating oligodendrocytes (for review see [7]). Mice lacking both LXRα and LXRβ display thinner myelin sheaths and reduced expression levels of myelin genes in the cerebellum. Furthermore, addition of pharmacological activators of LXRs to mixed glial cell cultures promotes oligodendroglial differentiation [8].

The nuclear receptor farnesoid-X-receptor (FXR; NR1H4) is expressed not only in the liver, gut, kidney and adipose tissue, but also in the immune cells [9, 10]. FXR heterodimerizes with other nuclear receptors, such as RXRs [11, 12]. The best-characterized endogenous FXR ligands are lipophilic bile acids, namely cholic acid and chenodeoxycholic acid. In addition, the synthetic compound GW4064 serves as an agonist with high receptor specificity and efficacy [11, 13, 14]. In accordance with its expression pattern, FXR activation has been shown to confer protection in several animal models of innate intestinal and hepatic inflammation [11]. In experimental autoimmune encephalomyelitis (EAE), an animal model of multiple sclerosis (MS), pharmacological activation of FXR significantly ameliorated the disease course by induction of anti-inflammatory macrophages [15, 25]. Interestingly, FXR agonists are currently tested in clinical trials for treatment of alcoholic hepatitis, type 2 diabetes mellitus and primary biliary cirrhosis, demonstrating that FXR already represents an attractive pharmacological target in human metabolic diseases. The beneficial effect of FXR agonists in EAE suggests that FXR might represent a potential target in inflammatory-demyelinating CNS diseases, such as MS. Oligodendroglial lineage cells are not only a primary target in MS but also a prerequisite for remyelination, an endogenous repair mechanism. However, especially in chronic MS lesions, remyelination is limited at least partly due to a differentiation block inhibiting the differentiation of oligodendroglial progenitor cells (OPCs) into myelinating oligodendrocytes [16–18]. Importantly, remyelination failure contributes to axonal damage and worsening of clinical signs in demyelinating animal models [19, 20].

Given its recently illustrated relevance in macrophage-mediated protection from CNS autoimmunity and the functional role of several nuclear receptors for oligodendroglial differentiation, we addressed the functional relevance of FXR in different brain-resident cells, especially focusing on oligodendrocytes. Here, we show that FXR is expressed in murine oligodendrocytes. However, lack or activation of FXR did not influence oligodendroglial differentiation in vitro or in vivo. Furthermore, FXR activation did not affect key immune functions of astrocytes and microglial cells. These data demonstrate that, in contrast to other nuclear receptors, FXR is dispensable for oligodendroglial differentiation as well as for brain-resident astrocytes and microglia and hence suggest that pharmacological activation of FXR to downregulate CNS inflammation does not interfere with repair mechanisms, such as remyelination.

## Materials and methods

### Animals

FXR Ko mice [21] were purchased from Jackson Laboratory and C57Bl/6 wild-type control mice from the animal facility in Münster, Germany. Animal handling and experiments were conducted according to the German Animal Welfare Act and approved by the responsible governmental authorities (LANUV Nordrhein-Westfalen; AZ-8.84.-02.05.20.12.286, 84.-02.04.2013.A029, AZ 84-02.04.2014.A221, AZ 84-02.04.2013.A374).

### Primary oligodendroglial culture and treatment

Primary OPCs were isolated using the immunopanning method as described earlier [22, 23]. OPCs/differentiating oligodendrocytes were incubated with 1 or 10 μM GW4064 (Tocris, 2473) for 24/48 h. Cell viability upon treatment was measured according to manufacturer’s protocol using CellTiter-Glo® Luminescent Cell Viability Assay (Promega, G7570). When treated with bone marrow-derived macrophage (BMM) supernatant, oligodendrocytes were incubated with 50% supernatant in culture medium for 48 h.

### Cerebellar slice cultures and treatment

Sagittal, cerebellar slices from P1 mice were dissected as described earlier [23]. After 12 days in culture, demyelination was induced by 16-h incubation with 0.5 mg/ml lysolecithin. During subsequent remyelination, the brain slices were incubated with 10 or 20 μM GW4064 for 14 days. The ex vivo slices were fixed and stained with anti-neurofilament 70 kDa (NFL; Dako, 1:500) and anti-MBP (Abcam, ab7349, 1:200). Confocal *z*-stacks of whole slices were acquired with a Zeiss LSM 700 confocal microscope, and images were analysed using NIH ImageJ. The extent of myelination was quantified by determining the ratio between MBP and NFL staining. Two independent experiments were performed, and in each experiment, six brain slices and three z-stacks from six brain slices were analysed for each concentration of GW4064 or DMSO.

### Macrophage and microglia culture and treatment

Preparation of bone marrow-derived macrophages and generation of primary astrocyte cultures via mixed glial culture preparation were performed as described previously [2]. The murine embryonic stem cell-derived microglial precursor cells (ESdMs) were provided by Harald Neumann (University of Bonn) and cultured according to their publication [2, 24]. Depending on the individual cell type, cells were treated with different concentrations of the synthetic FXR agonist GW4064 (Tocris, 2473); concentrations depended on individual cell type. Control groups were treated with the equivalent volume of solvent, i.e. DMSO. ESdMs and primary astrocytes were incubated for 24 h with the BMM-conditioned supernatant, before stimulation with LPS and IFNγ.

### Generation of BMM supernatant

To produce BMM-conditioned supernatant, BMMs were treated with 15 μM GW4064 or DMSO and cultured for 72 h [25].

### Detection of TNFα and nitric oxide

TNFα in cell culture supernatants was quantified using ELISA Ready-SET-Go!® (murine TNFα; eBioscience) according to the manufacturer’s recommendations. Nitrite concentrations were measured in the supernatants using the Griess Reagent Kit (Invitrogen) according to the manufacturer’s instructions.

### RT^2^ Profiler™ PCR Array

Treated ESdMs and astrocytes were stimulated with LPS and INFγ for 6 h. RNA isolation was performed with RNeasy Mini Kit (Qiagen) combined with the RNase-Free DNase Set (Qiagen) to digest DNA. For transcription to complementary DNA (cDNA), the RT^2^ FirstStrand Kit (Qiagen) was utilized. Afterwards, the RT^2^ Profiler™ PCR Array Inflammatory Response & Autoimmunity (#PAMM-077Z; Qiagen) was performed with RT^2^ Real-Time SYBR Green PCR Master Mix (SuperArray Bioscience) according to the manufacturer’s protocol. For normalization, the housekeeping gene *Hsp90ab1* was taken as internal control. Analysis was performed at the RT^2^ Profiler PCR Array Data Analysis Web portal (provided from Qiagen).

### RNA isolation and qRT-PCR

Total RNA from cells was isolated using peqGOLD Total RNA Kit (12-6634, PeqLab Biotechnologie GmbH). Messenger RNA (mRNA) was transcribed into cDNA by reverse transcription reaction (High Capacity cDNA Transcription Kit, Applied Biosystems), and cDNA was diluted to a final concentration of 0.75 ng/μl. qRT-PCR was performed using Power SYBR® Green PCR Master Mix (Applied Biosystems) and StepOne Plus real-time cycler (Applied Biosystems). The following primers were used: *Fxr* fw: GCCACAGATTTCCTCCTCGT, *Fxr* rev: CAGTCTCTCCCTGGTACCCA, *Mbp* fw: GTACAAGGACTCACACACGAGA, *Mbp* rev: GTTCGAGGTGTCACAATGTTCT, *RPLP0* fw: CGACCTGGAAGTCCAACTAC and *RPLP0* rev: ATCTGCTGCATCTGCTTG, and data were normalized using *RPLP0* as internal control.

### Immunocytochemistry (ICC)

OPCs were fixed directly after seeding or differentiated and fixed after 48 h as mature oligodendrocytes. Cells were permeabilised for 10 min in 0.5% Triton X-100 in PBS and blocked using 5% FCS/PBS for 30 min. The primary antibodies were rat anti-MBP (Abcam, ab7349, 1:200), rabbit anti-PDGFRα (Santa Cruz, SSC338, 1:300), rabbit-FXR NR1H4 (Abcam, ab28676, 1:200; or Santa Cruz sc-13063, 1:100) and mouse anti-Olig2 (Medac, 387M-16, 1:200). Incubation was performed at 4 °C over night. Secondary antibody staining was performed using Cy™3 Anti-Rat (1:500) (Jackson, 112-165-167) and anti-Rabbit Alexa Fluor® 488 conjugate (1:500) (Jackson, 111-545-144) for 2 h at RT before embedding with Roti®-Mount FluorCare DAPI (Carl Roth, HP20.1). Images were taken using the laser scanning microscope (LSM 700, Zeiss Jena). At least 200 cells were quantified, and the numbers of MBP+ and PDGFRα+ cells were assessed as percentage of total DAPI+ cells.

### Immunohistochemistry (IHC)

Ten-day and 8-week-old WT or FXR Ko mice were sacrificed and intracardially perfused. The spleens, spinal cords and brains were removed and fixed in 4% PFA overnight. Paraffin sections (4 μm) were pretreated with citrate buffer (pH 6) and stained using an automated immunostainer (AutostainerLink 48, Dako). Primary antibodies were specific to FXR (NR1H4, rabbit, Abcam ab28676 1:200; or Santa Cruz sc-13063, 1:50), NogoA (mouse, 11c7, a generous gift from M.E. Schwab, Brain Research Institute, University of Zürich and Department of Biology, Swiss Federal Institute of Technology Zürich, Switzerland, 1:15,000) and Olig2 (rabbit, 18953, IBL, 1:150 and mouse, 387M-16, Medac, 1:200). Numbers of oligodendroglial cells were quantified in a blinded fashion in the corpus callosum, cerebellum and spinal cord in standardized microscopic fields of 10,000 μm^2^ each defined by an ocular morphometric grid.

### Statistics

All cell culture experiments were performed in triplicates and replicated at least three times. All statistical analyses were performed using GraphPad Prism 5.03 (GraphPad Software Inc., San Diego, CA). In text and figures, results are provided as mean ± SEM. Multiple comparisons in the same data set were analysed by the Bonferroni-corrected (for selected groups) one-way ANOVA tests. Single comparisons to control were made using two-tailed Student’s *t* test. *p* values <0.05 were considered significant.

## Results

### FXR is expressed in oligodendrocytes

**Fig. 1:**
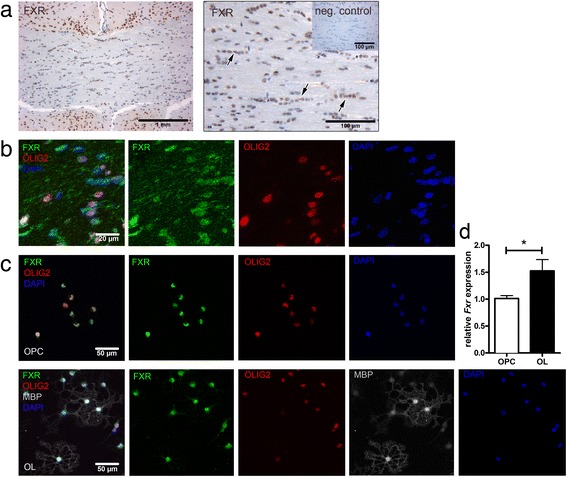
FXR is expressed by oligodendroglial cells in vivo and in vitro. Immunohistochemical analysis revealed oligodendroglial cells in the corpus callosum of adult mice express FXR protein. Oligodendrocytes were identified by their linear arrangement (*arrows*) and coexpression of OLIG2, respectively (**a**, **b**). In primary, murine oligodendroglial cells, FXR is expressed in OLIG2+ OPCs and mature oligodendrocytes, identified by OLIG2 and MBP expression after 48 h of in vitro differentiation (**c**). The relative *Fxr* gene expression level is increased in mature oligodendrocytes compared to OPC (**d**), *n* = 3; two-tailed Student’s *t* test, *p < 0.05; all images are representative

Histological analysis of brain sections from adult mice revealed expression of FXR in oligodendroglial lineage cells, which were identified by their typical linear arrangement within the corpus callosum (Fig. 1a (arrows)) and by coexpression with OLIG2 (Fig. 1b). Additionally, we analysed expression of FXR in oligodendrocytes in vitro. Before induction of differentiation using CNTF and NT3 [22], almost 100% of the oligodendroglial lineage cells express PDGFRα, a marker specifically expressed by OPCs. After 24 h of differentiation, approximately 15% of the cells express MBP, a marker of mature oligodendrocytes; this percentage increases to 35% after 48 h, whereas PDGFRα+ OPCs account for approximately 10% of the cells. FXR protein expression was detected in OPCs and mature oligodendrocytes using immunocytochemistry (Fig. 1c). Quantification of *Fxr* gene expression demonstrated an increase in *Fxr* mRNA expression during oligodendroglial differentiation (Fig. 1d).

### Lack of FXR does not impair oligodendroglial differentiation in vivo or in vitro

**Fig. 2:**
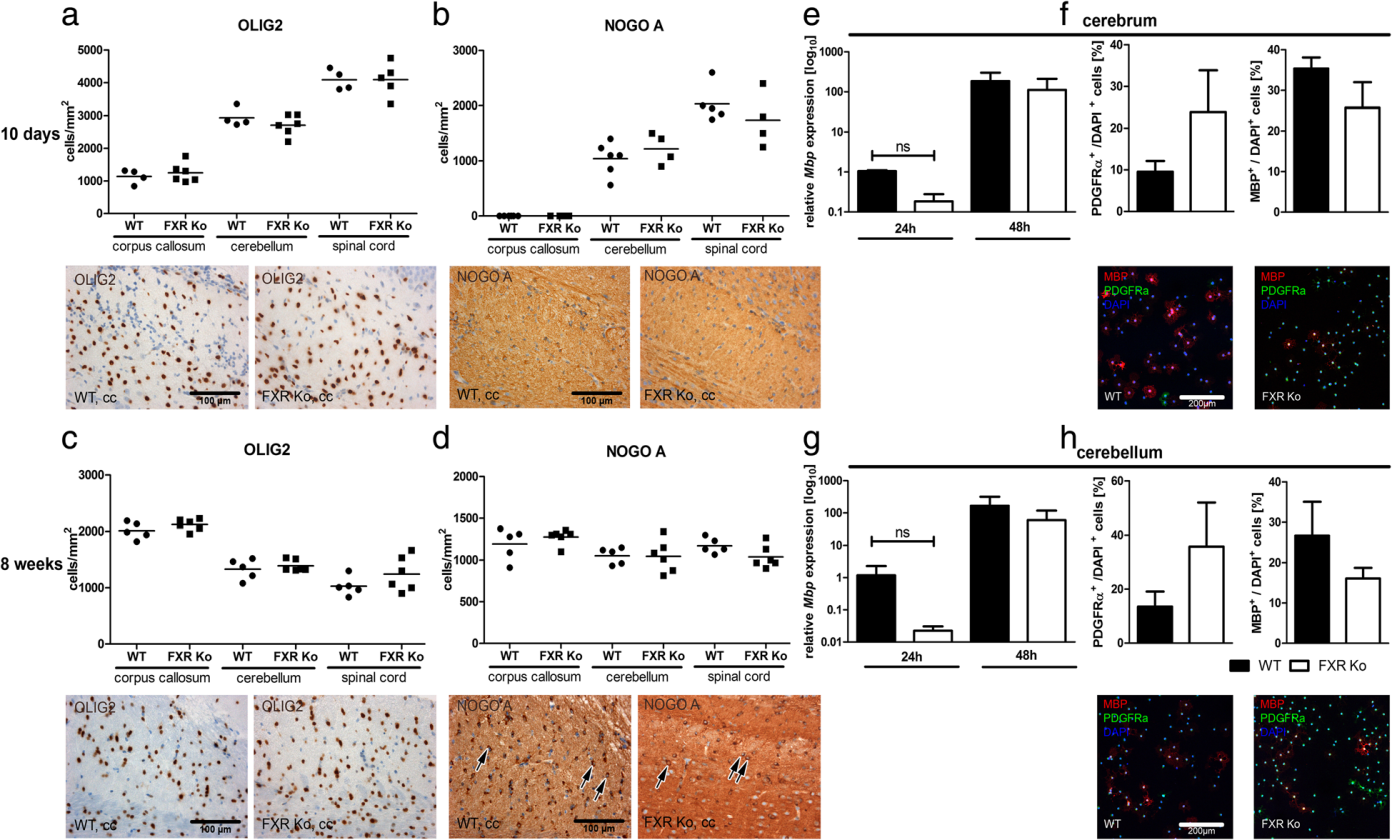
Loss of FXR does not impact oligodendroglial differentiation in vitro and in vivo. Immunohistochemical staining of WT and FXR Ko mice, either 10 days or 8 weeks old, display no differences in the number of OLIG2+ oligodendroglial cells in the corpus callosum (cc), cerebellum or spinal cord (**a**, **c**). In addition, the proportion of mature NOGO A+ oligodendrocytes (**b**, **d**) is not altered in FXR Ko mice compared to WT mice. In vitro cultured, primary oligodendrocytes isolated either from the cerebrum (**e**, **f**) or cerebellum (**g**, **h**) display no significant difference in the *Mbp* gene expression (**e**, **g**) after 24 and 48 h of differentiation or the percentage of PDGFRα+ OPCs or mature MBP+ oligodendrocytes (**f**, **h**) after 48 h of differentiation. In vivo: number of cells/mm^2^ was determined using an ocular grit, *n* = 4–6 animals, two-tailed Student’s *t* test; in vitro: *n* = 3, one-way ANOVA with Bonferroni’s correction, 200 cells per condition and two-tailed Student’s *t* test, ns >0.05; all images are representative

Utilizing FXR Ko mice, we determined the impact of FXR loss on the numbers of oligodendrocytes 10 days and 8 weeks after birth. To determine the numbers of OPCs and mature oligodendrocytes, we used OLIG2, a transcription factor that is expressed in both cell populations. Quantification of OLIG2+ cells in the corpus callosum, cerebellum and spinal cord of FXR Ko and WT mice did not reveal differences between the two genotypes (Fig. 2a, c). Additionally, the number of mature NogoA positive oligodendrocytes was not affected by loss of FXR in any of the analysed CNS regions (Fig. 2b, d). The differentiation of FXR Ko or WT oligodendrocytes isolated from either cerebrum or cerebellum was examined in vitro (Fig. 2e–h). After 24 h of differentiation, *Mbp* expression levels were slightly reduced in FXR Ko cells; however, no significant alteration in gene expression could be observed in either cerebral or cerebellar oligodendrocytes (Fig. 2e, g). In addition, no difference in the amount of PDGFRα+ OPCs or MBP+ mature oligodendrocytes could be detected comparing FXR Ko and WT cells (Fig. 2f, h). In summary, lack of FXR had no major effect on the numbers of oligodendrocytes or oligodendroglial differentiation, neither in vivo nor in vitro.

### Activation of FXR does not affect oligodendroglial differentiation

**Fig. 3:**
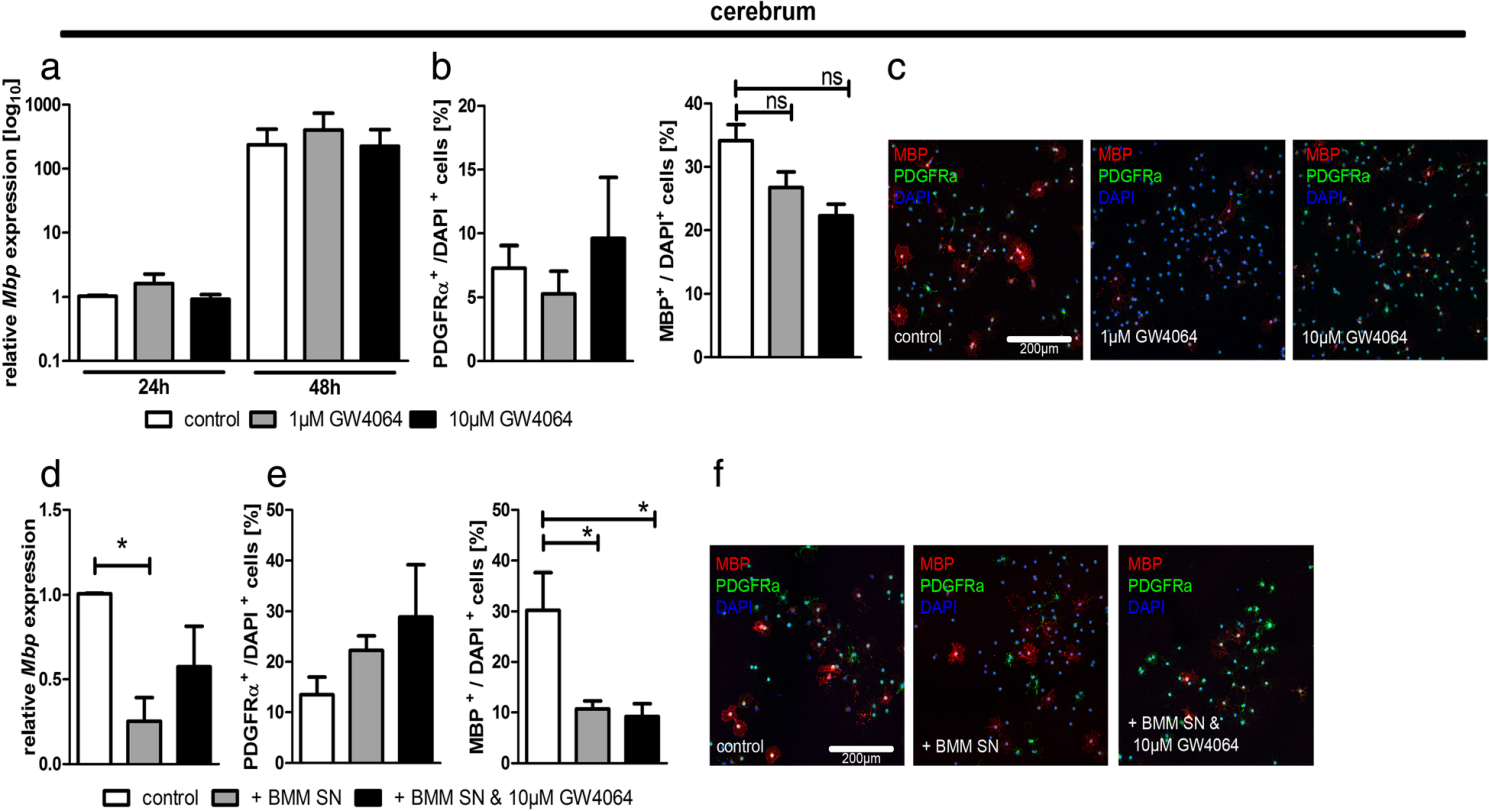
Cerebral oligodendroglial differentiation is not affected by direct FXR activation or supernatant of FXR-activated BMMs. Cerebral oligodendrocytes show no differences in *Mbp* expression when incubated for 24 or 48 h with 1 μM or 10 μM GW4064 compared to control cells treated with solvent (**a**). Numbers of PDGFRα+ OPCs and MBP+ oligodendrocytes are not altered due to GW4064 treatment after 48 h (**b**, **c** ). Supernatants of BMMs reduce *Mbp* gene expression (**d**) and the percentage of MBP+ oligodendrocytes after 48 h of differentiation, but this effect is independent of GW4064 (**e**, **f** ). Percentages of PDGFRα+ OPC are not affected (**e**, **f**). *n* = 3, one-way ANOVA with Bonferroni’s correction, 200 cells per condition, ns >0.05; *p < 0.05; all images are representative

OPCs isolated either from the cerebrum or from the cerebellum were treated with 1 or 10 μM of the pharmacological FXR agonist GW4064 after induction of differentiation for up to 48 h. GW4064 treatment did not affect the viability of the oligodendroglial cells (data not shown). mRNA expression levels of the myelin-associated gene *Mbp* increased over time, but no differences in the relative expression levels were detected between GW4064-treated and control-treated oligodendrocytes (Fig. 3a). Using immunocytochemistry, we determined the number of PDGFRα+ OPCs and MBP+ mature oligodendrocytes after 48 h of GW4064 treatment. The addition of GW4064 to oligodendroglial cell cultures did not result in a significant change in the percentage of PDGFRα+ OPCs or MBP+ mature oligodendrocytes (Fig. 3b, c). Additionally, we analysed the impact of GW4064 application to oligodendroglial cells isolated from the cerebellum; however, no alterations in *Mbp* gene expression or changes in the percentage of PDGFRα+ OPCs and MBP+ mature oligodendrocytes compared to controls were observed (Additional file 1: Figure S1a–c). These data show that direct activation of FXR has no impact on oligodendroglial differentiation.

### Remyelination following toxic-induced demyelination in cerebellar brain cultures is not impacted by activation of FXR

To analyse a potential impact of FXR activation on the process of remyelination, we made use of cerebellar slice cultures that were demyelinated by lysolecithin. We applied two different concentrations of GW4064 to demyelinated cerebellar slice cultures during 14 days of remyelination. Evaluation of the amount of MBP+ and NFL+ axons revealed no influence of GW4064 treatment on the remyeliation capacity of oligodendrocytes after toxic demyelination in this ex vivo model (Additional file 1: Figure S1g, h).

### No indirect effect of FXR-activated macrophages on oligodendroglial differentiation

Since peripheral macrophages migrate into the CNS during MS pathogenesis and then modulate the inflammatory activity of brain-resident cells and given the strong immune-modulatory properties of FXR in macrophages [25], we next addressed whether FXR might indirectly affect oligodendrocytes via FXR-modulated macrophages. To this end, bone marrow-derived macrophages (BMM) were cultured and treated with 10 μM GW4064 to activate FXR [25]. Subsequently, OPCs isolated from the cerebrum were cultured in the presence of supernatants from GW4064-exposed BMMs to analyse the effect on oligodendroglial differentiation. Controls included oligodendroglial lineage cells cultured either in the presence of supernatants from BMM without GW4064 exposure or without any BMM supernatants. After 48 h of differentiation, *Mbp* expression levels and numbers of mature MBP+ oligodendrocytes were significantly reduced in cerebral oligodendrocytes exposed to BMM supernatants; however, this was independent of GW4064 treatment (Fig. 3d–f). No significant differences in the numbers of PDGFRα+ OPCs were observed in cultures exposed to BMM-conditioned medium with or without GW4064 or controls (Fig. 3e, f). Cerebellar oligodendrocytes showed no significant alteration of *Mbp* gene expression, but a significant decrease in MBP+ mature oligodendrocytes after addition of BMM supernatants compared to controls without BMM supernatants; however, conditioning with GW4064 had no additional effect on oligodendroglial differentiation (Additional file 1: Figure S1d–f). Furthermore, no significant differences in the numbers of PDGFRα+ OPCs were observed (Additional file 1: Figure S1e, f).

### FXR activation does not affect immune functions of brain-resident astrocytes and microglia

**Fig. 4:**
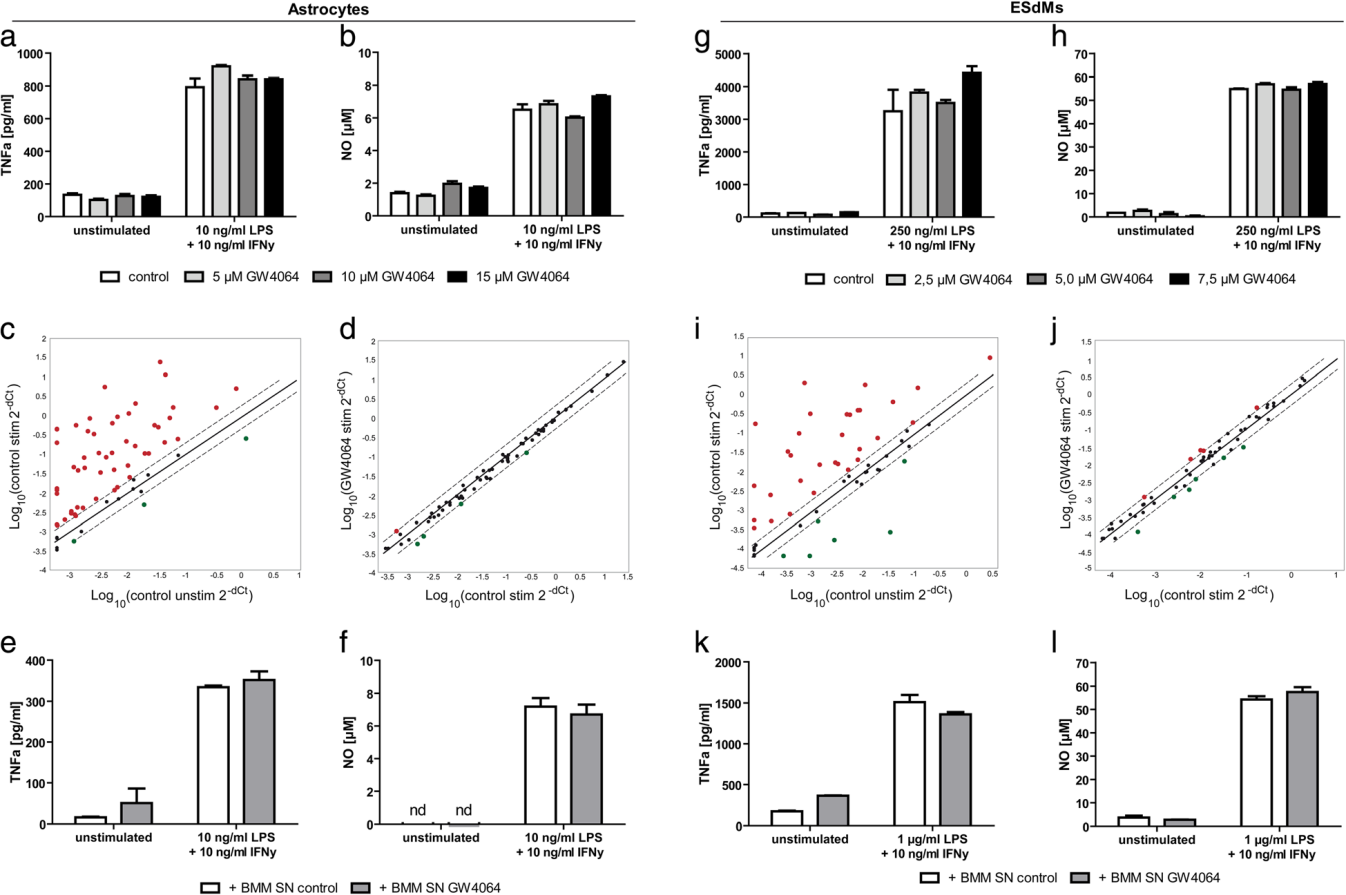
Immune functions of brain-resident astrocytes and microglia are not affected by direct FXR activation or supernatant of FXR-activated BMMs. The direct treatment with GW4064 (**a**, **b**, **d**, **g**, **h**, **j**) and the incubation with conditioned BMM supernatant of astrocytes (**e**, **f**) and ESdMs (**k**, **l**) do not suppress secretion of TNFα and NO. ESdMs and primary astrocytes were treated with different concentrations of GW4064 for 7 days. RNA was isolated after 6 h of stimulation with LPS and IFNγ. Differential expression of genes was assessed by performing RT^2^ Profiler PCR Assay (**c**, **d**, **i**, **j**). After stimulation, a strong differential expression of genes in astrocytes (**c**) and ESdM (**i**) compared to unstimulated cells was detectable. However, treatment with GW4064 had no influence on the gene expression of either astrocytes (**d**) or ESdMs (**j**). Supernatants of pretreated BMMs were collected after 3 days of culturing and were transferred to ESdMs or astrocytes. Secreted amount of TNFα was determined by ELISA after stimulation with LPS and IFNγ for 24 h (**g**, **k**: ESdMs) or 72 h (**a**, **e**: astrocytes). Concentration of produced NO was detected by Griess assay after 48 h of stimulation of ESdMs (**h**, **l**) and after 72 h in the supernatant of astrocytes (**b**, **f**). Bar graphs: data are represented as mean ± SEM. Shown is one representative experiment of at least three independent experiments with triplets for each condition, two-tailed Student’s *t* test. Scatter plots: data are represented as logarithmic average fold change. Shown are data from two biological replicates

In light of the described anti-inflammatory properties of FXR on macrophages [25], we wondered whether pharmacological FXR activation might suppress inflammatory activity of brain-resident astrocytes or microglia, which could also indirectly influence oligodendrocyte differentiation and function in local inflammatory processes during CNS autoimmunity. However, FXR activation in primary astrocytes by different concentrations of GW4064 did not affect the production of key pro-inflammatory molecules TNFα (Fig. 4a) and NO (Fig. 4b). In this line, also activation of FXR in embryonic stem cell-derived microglial cells (ESdMs; [24]) did not suppress TNFα (Fig. 4g) and NO (Fig. 4h) production. Furthermore, no differences in the expression of 84 genes involved in inflammatory immune responses, such as genes of cytokines, interleukins and chemokines, were observed in DMSO-treated cells compared to GW4064-treated astrocytes (Fig. 4c, d) and ESdMs (Fig. 4i, j), respectively.

### Supernatant of GW4064-treated BMMs does not affect astrocytic and microglial immune functions

We next investigated the influence of conditioned supernatant from FXR-activated BMMs on astrocytes and microglial cells. However, there was no modulation of TNFα (Fig. 4e, k) or NO (Fig. 4f, l) production detectable in astrocytes as well as in microglial cells upon incubation with conditioned supernatants. In conclusion, these data indicate that FXR does not play a major role in controlling pro-inflammatory activities of brain-resident cells, neither directly nor indirectly by modulating inflammatory macrophage activity.

## Discussion

Here, we demonstrate that FXR is expressed by OPCs and mature oligodendrocytes, like microglia and astrocytes [25]. However, lack of FXR did not affect oligodendroglial differentiation in vitro or in vivo. Furthermore, activation of FXR using the synthetic agonist GW4064 did not affect oligodendroglial differentiation, ex vivo remyelination or pro-inflammatory activation of astrocytes or microglia.

Several nuclear receptors, such as RXRα, RXRβ, RXRγ, VDR, TR, LXRα and LXRβ, are expressed by oligodendrocytes and have been shown to affect oligodendroglial function [5, 6, 8, 26]. Lack of TR or LXRα/LXRβ results in delayed oligodendroglial differentiation or hypomyelination in the cerebellum, respectively [8, 26]. Similarly, blocking and/or downregulation of VDR, TRs or RXRγ impaired oligodendroglial differentiation [5, 27]. In contrast, our results provide no indication that lack of FXR delays oligodendroglial differentiation in vitro or in vivo. FXR forms a heterodimer with RXR to induce expression of target genes, but FXR can also function as a repressor by suppressing gene expression [13]. Our results suggest that, at least in oligodendrocytes, the function of FXR is redundant to or is compensated by other nuclear receptors.

Activation of several nuclear receptors such as RXRγ, TR, VDR and LXRα/LXRβ promotes oligodendroglial differentiation [3, 5–8]. This is in contrast to our results, which show that activation of FXR by the synthetic agonist GW4064 had no effect on the number of MBP+ mature oligodendrocytes or expression levels of MBP. Oligodendrocytes display a region-dependent heterogeneity within the CNS [28], and interestingly, lack of LXRα/β is associated with hypomyelination in the cerebellum [8]. To determine whether FXR might play a more prominent role in the differentiation of cerebellar oligodendrocytes, we repeated our in vitro experiments using oligodendrocytes isolated specifically from the cerebellum. However, neither lack nor activation of FXR modulated the differentiation of cerebellar oligodendrocytes. Likewise, addition of FXR agonists to demyelinated cerebellar slice cultures did not exhibit an effect on remyelination in this ex vivo model in which exclusively CNS-resident cells are present.

It has recently been shown that activation of FXR in BMM induced an anti-inflammatory phenotype characterized by downregulation of pro-inflammatory and induction of anti-inflammatory genes [25]. In light of the finding by Miron and colleagues who demonstrated an increased differentiation of rodent oligodendrocytes cultured in the presence of supernatants derived from anti-inflammatory microglial cells, we hypothesized that FXR-activated BMM might indirectly modulate oligodendrocyte differentiation [29]. However, our results provide no evidence that activation of FXR in macrophages has a similar, indirect effect on oligodendroglial differentiation. Oligodendroglial differentiation and remyelination can also be accelerated in vivo by improved clearance of myelin debris by macrophages as it has been shown for the RXR agonist bexarotene [30]. Whether activation of FXR in macrophages may potentially result in increased myelin phagocytosis is currently unknown. However, this could indirectly contribute to oligodendrocyte differentiation and remyelination.

The anti-inflammatory phenotype induced by GW4064-mediated activation of FXR in peripheral myeloid cells, such as BMMs, is well documented [15, 25]. However, expression of FXR is not limited to myeloid cells, but is also present in microglia as well as astrocytes [25]. Interestingly, FXR activation in microglia using GW4064 did not result in downregulation of pro-inflammatory key molecules, such as TNFα and NO, or altered expression of pro- and anti-inflammatory genes encoding for cytokines, interleukins or chemokines as well as their receptors. These data illustrate that activation of FXR via GW4064 has no influence on the immune responses of microglia and astrocytes. This contrasts with the activation of other nuclear receptors such as LXR, RXR and PPARα [31, 32]. Considering previously published data [25], our results suggest that FXR plays a pivotal role in downregulation of pro-inflammatory molecules in peripheral myeloid cells, but not in CNS-resident microglia suggesting heterogeneity between the two cell populations despite overlapping functions. This may be due to a considerable diversity of gene expression patterns between tissue macrophages from different mouse organs [33] and could further explain the recent observation that blood-derived monocytes might mainly initiate demyelination in EAE whereas microglia potentially primarily clear myelin debris [34].

## Conclusion

In summary, we demonstrated that activation of FXR by GW4064 does not modulate oligodendroglial differentiation directly or indirectly via FXR activation in macrophages. Furthermore, there is no indication that GW4064 directly or indirectly influences the pro-inflammatory profiles of CNS-resident astrocytes or microglia. These data suggest that FXR agonists, such as GW4064, represent a potential therapeutic approach for MS which specifically targets peripheral immune cells including macrophages, but not brain-resident cells, such as oligodendrocytes, astrocytes or microglia.

## Additional file

Additional file 1: Figure S1. Cerebellar oligodendroglial differentiation is not affected by direct FXR activation or supernatant of FXR-activated BMMs. Addition of GW4064 during the differentiation of cerebellar oligodendrocytes over 24 and 48 h does not influence *Mbp* expression levels (a) or the percentages of PDGFRα+ OPCs or MBP+ oligodendrocytes (b, c). Oligodendrocytes of cerebellar origin exhibit no significant difference in their *Mbp* expression level when incubated with supernatants from BMM cultured either in the presence or absence of GW4064 (d). The percentage of OPCs is unchanged after incubation with BMM-conditioned medium; the percentage of MBP+ oligodendrocytes is reduced independent of additional GW4064 treatment (e, f). Activation of FXR cultures using 10 and 20 μM GW4064 during 14 days of remyelination in cerebellar slice after toxic demyelination does not alter the ratio of MBP+ and NFL+ axonal fibres (g). Remyelinated fibres are exemplarily highlighted (arrows). Note that a high amount of MBP+ debris is still present after 14 days of remyelination (h). In vitro*: n* = 3, 1way ANOVA with Bonferroni’s correction, 200 cells per condition were evaluated, **p* < 0.05; ex vivo*: n* = 2, 1way ANOVA with Bonferroni`s correction, 6 slices with 3 images each per condition and preparation; all images are representative. (TIF 5564 kb)

## Abbreviations

ANOVA: Analysis of variance
BMM: Bone marrow-derived macrophage
CNS: Central nervous system
CNTF: Ciliary neurotrophic factor
DAPI: 4′,6-Diamidin-2-phenylindol
DMSO: Dimethylsulfoxid
EAE: Experimental autoimmune encephalomyelitis
ELISA: Enzyme-linked immunosorbent assay
ESdMs: Embryonic stem cell-derived microglial precursor cells
fw: Forward
FXR: Farnesoid-X-receptor
Hsp90ab1: Heat shock protein 90 kDa alpha family class B member 1
ICC: Immunocytochemistry
ICH: Immunohistochemistry
IFNγ: Interferon gamma
Ko: Knock out
LPS: Lipopolysaccharide
LSM: Laser scanning microscope
LXR: Liver X receptor
MBP: Myelin basic protein
mRNA: Messenger RNA
MS: Multiple sclerosis
NFL: Neurofilament 70 kDa
NO: Nitric oxide
NogoA: Neurite outgrowth inhibitor A
NR1H4: Farnesoid-X-receptor
NT3: Neurotrophin-3
OL: Mature oligodendrocyte
Olig2: Oligodendrocyte transcription factor
OPC: Oligodendroglial progenitor cell
PDGF: Platelet-derived growth factor
PDGFRα: Platelet-derived growth factor receptor alpha
PFA: Paraformaldehyde
PPAR: Peroxisome proliferator-activated receptor
qRT-PCR: Quantitative real-time polymerase chain reaction
rev: Reverse
*RPLP0*: 60S acidic ribosomal protein P0
RXR: Retinoid X receptor
SEM: Standard error of the mean
T3: Triiodothyronine
TNFα: Tumor necrosis factor alpha
TR: Thyroid receptor
VDR: Vitamin D receptor
WT: Wild type
