# Supplementary figures and images for: Activation of FXR pathway does not alter glial cell function
Source: J Neuroinflammation. 2017 Mar 28;14:66. doi: 10.1186/s12974-017-0833-6 (PMC5371249; doi:10.1186/s12974-017-0833-6)

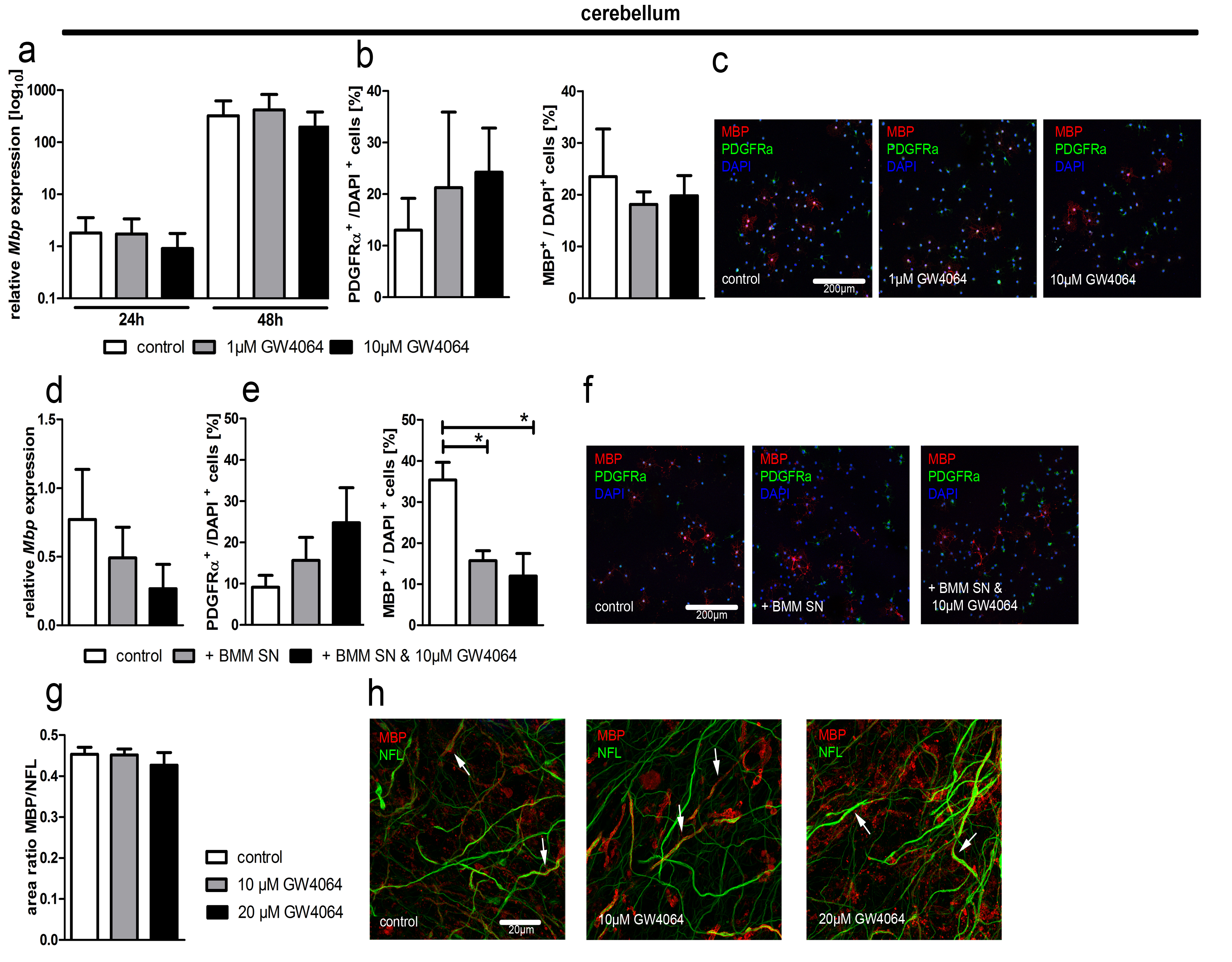

Supplement: Additional file 1: Figure S1. — Cerebellar oligodendroglial differentiation is not affected by direct FXR activation or supernatant of FXR-activated BMMs. Addition of GW4064 during the differentiation of cerebellar oligodendrocytes over 24 and 48 h does not influence Mbp expression levels (a) or the percentages of PDGFRα+ OPCs or MBP+ oligodendrocytes (b, c). Oligodendrocytes of cerebellar origin exhibit no significant difference in their Mbp expression level when incubated with supernatants from BMM cultured either in the presence or absence of GW4064 (d). The percentage of OPCs is unchanged after incubation with BMM-conditioned medium; the percentage of MBP+ oligodendrocytes is reduced independent of additional GW4064 treatment (e, f). Activation of FXR cultures using 10 and 20 μM GW4064 during 14 days of remyelination in cerebellar slice after toxic demyelination does not alter the ratio of MBP+ and NFL+ axonal fibres (g). Remyelinated fibres are exemplarily highlighted (arrows). Note that a high amount of MBP+ debris is still present after 14 days of remyelination (h). In vitro: n = 3, 1way ANOVA with Bonferroni’s correction, 200 cells per condition were evaluated, *p < 0.05; ex vivo: n = 2, 1way ANOVA with Bonferroni`s correction, 6 slices with 3 images each per condition and preparation; all images are representative. (TIF 5564 kb) [file 12974_2017_833_MOESM1_ESM.tif]
